# Medical Items

**Published:** 1897-07

**Authors:** 


### *MEDICAL ITEMS.*

We regret to learn of the illness of Dr. E. A. Cobleigh, of Chattanooga.

The Plant System Hospital, at Waycross, Ga., has been completed, at a cost of $45,000.

Dr. Edward N. Liell, late lecturer on gynecology in the New York Polyclinic, has moved to Jacksonville, Fla.

A fond mother reported that with the aid of the X rays a coin which her son had swallowed had been distinctly located in his sarcophagus.—*Ex.*

First Deaf-Mute (speaking by finger-signs): What makes you stutter so? Second Deaf-Mute (speaking ditto): I can't help it. I fell off my bicycle yesterday and sprained my first finger.—*Judge.*

Professor Charles S. Briggs has resigned the Professorship of Surgery in the medical department of the University of Nashville, on account of "dissatisfaction with the reigning policy of the school."

Died:—Dr. R. J. Goodman, of Sparks, Ga., June 16th; Dr. John Norwood, of Columbus, Ga., June 28th, aged 61; Dr. J. W. Drewry, of Eufaula, Ala., June 21st, aged 70; Dr. P. M. Tidwell, of Fairburn, Ga., June 29th, aged 73. Dr. Tidwell was one of the pioneer citizens of Fairburn and had practiced medicine there since 1853.

Menander said, that all diseases were curable by sleep—a broad statement, in which, nevertheless, there may be something that is true, for good sleepers are ever, as I think, the most curable patients; and I would always rather hear a sick person had slept, than had taken regularly the prescribed medicine during sleeping hours. —*Sir Benj. Ward Richardson.*

---

Who will answer this?

An anonymous correspondent says that a patient asked him this question: "Can a young woman who expected to be a (God) mother of a child, and who was disappointed because the child did not come, be properly said to have had a miscarriage?" The question was based upon a recent occurrence in a Georgia city.

---

Must the oyster go, too? A writer in *Modern Medicine* says: "It is gratifying to know that that filthy bivalve, the oyster, whose proper function in the world is the consumption of the ooze and slime which cover the bottom of the ocean, extending to submarine plants, is rapidly creating such a bad reputation for itself that there is already a prospect that its consumption as an article of food may be practically abandoned."

---

The *Medical Examiner* (New York), is an exceedingly valuable publication for doctors doing much life insurance work. Examinations for life insurance form an important part of the labors of many physicians, and anything which can aid them in the discharge of these particular duties, will be, or should be, welcome. The *Medical Examiner* always contains articles and suggestions of such a nature; more than any other publication that we now recall. It is a decided success in its special field.

We have received the ninth volume of Transactions of the Southern Surgical and Gynecological Association, for the meeting held in Nashville, November, 1896. The volume is increased in size over former editions, and contains a large number of interesting and valuable surgical and gynecological papers. The president of the Association is Dr. Geo. B. Johnston, of Richmond, the secretary, Dr. W. E. B. Davis, of Birmingham. Next meeting will be held in St. Louis, November 9th to 12th.

---

Following are the officers of the Alabama Medical Association for the coming year: President, Dr. L. L. Hill, of Montgomery; Senior Vice-President, Dr. John C. LeGrand, of Anniston; Junior Vice-President, Dr. E. L. Marechal, of Mobile; Councilors for five years, Drs. Seeley, of Montgomery, and Sanders of Mobile, both succeeding themselves. Councilor to fill the unexpired term of Dr. Jerome Cochran, Dr. J. P. Furniss, of Selma. The Association will meet next year in Birmingham.

---

A fairy story written by a young lady of six years is just out. The fairies in the book, like other mortals, become ill and require the services of the fairy physician, who discovers that one has scarlet fever, another diphtheria, and still another typhoid fever. The fairy mother is told all about microbes, etc., and is instructed to boil the water. She does not understand. If the microbe has the fever, why does not the fever, which kills the little fairies, kill the microbes? And if the microbe does not have the fever, how can it give the fever? How can a thing give a thing that it does not have?—*Ex.*

---

We have just received the report of the board of health for the city of Atlanta for 1896. It shows 1,861 deaths from all diseases during the year—whites 823, colored 1,038. The annual mortality rate was about 22 per thousand, the estimated population being 50,000 white and 36,000 colored. The mortality rate was 16.4 per thousand for whites, and 28.8 for blacks. Consumption caused 226 deaths, or 11.11 per cent. Of these 81 were white and 145 colored. Consumption mortality for whites, 9.8 per cent.; for blacks, nearly 14 per cent. Suicides, four whites and one colored. Since 1880, the colored death-rate has gradually increased from 39.8 per cent. of the total morality to 55.7 per cent. in 1896.

---

If you've got a thought that's happy
Boil it down;
Make it short and crisp and snappy,—
Boil it down.
When your brain its coin has minted,
Down the page your pen has sprinted,
If you want your effort printed,
Boil it down.

Take out every surplus letter,—
Boil it down;
Fewer syllables the better,—
Boil it down.
Make your meaning plain—explain it,
So we'll know, nor merely guess it;
Then, my friend, ere you address it,
Boil it down. —*Ex.*

---

Is there a typho-malarial fever? Yes, in the brains of the doctors, but not in the bodies of the patients. There is no combined hybrid disease and it is only due to Woodward to say that he did not recognize a hybrid disease. Typho-malaria is a villainous name and should be banished from our vocabularies, and no doctor should ever use it, particularly to his patients. It gives a man a wrong sense of security and the doctor wastes a lot of good medicine, a lot of quinine, for instance, because he thinks there is some symptom that points to malaria. I am happy to say that cases of typho-malaria are disappearing slowly from the health reports; they ought to be banished entirely. Chills, as I told you, occur frequently at the outset of a disease and they may occur throughout the course of a typhoid fever. The State boards of health hereafter should return to every physician who sends in a diagnosis of typho-malaria his blank and ask for something better. It is too late in the day, gentlemen, to make that diagnosis.—*Dr. William Osler, in Maryland Medical Journal.*

---

Dr. Nicholas Senn, late President of the American Medical Association, offers a gold medal to the member of the Association who shall present the best essay upon some surgical subject. The award will be made under the following conditions: *a.* The name of the author of each competing essay shall be enclosed in a sealed envelope, bearing a suitable motto or device, the essay itself bearing the same motto or device. The title of the successful essay and the motto or device to be read at the meeting at which the award is made, and the corresponding envelope to be then and there opened and the name of the successful author announced. *b.* All successful essays become the property of the Association. *c.* The medal shall be conferred and honorable mention made of the two other essays considered worthy of this distinction, at a general meeting of the Association. *d.* The competition is to be confined to those who, at the time of entering the competition, as well as at the time of conferring the medal, shall be members of the American Medical Association. *e.* The competition for the medal shall be closed three months before the next annual meeting of the American Medical Association, and no essay will be received after March 1, 1898. The committee on awarding this medal will be: Dr. J. McF. Gaston, Atlanta, Ga.; Dr. H. O. Walker, Detroit, Mich.; and Dr. S. H. Weeks, Portland, Maine. Competitors should forward their essays to Dr. Gaston, chairman of the committee.

Some important forthcoming medical books are announced by the well-known publisher, Mr. W. B. Saunders, of Philadelphia. Among these are:

An American Text-Book of Genito-Urinary and Skin Diseases. Edited by L. Bolton Bangs, M.D., New York; and Wm. A. Hardaway, M. D., St. Louis.

An American Text-Book of Diseases of the Eye, Ear, Nose and Throat. Edited by G. E. de Schweinitz, M. D.; and B. Alexander Randall, M.D., Philadelphia.

Surgical Diagnosis and Treatment. By J. D. McDonald, M.D., Minneapolis.

Text-Book of the Theory and Practice of Medicine. By James M. Anders, M.D., Ph.D., LL.D., Philadelphia.

Tuberculosis of the Genito-Urinary Apparatus, Male and Female. By Nicholas Senn, M.D., Ph.D., LL.D., Chicago.

A Text-Book of Gynecology. By Charles B. Penrose, M.D., Professor of Gynecology, Philadelphia.

A Text-Book of Obstetrics. By Barton Cooke Hirst, M.D., Philadelphia.

Manual of Orthopedic Surgery. By James E. Moore, M.D., University of Minnesota.

Text-Book of Embryology. By John C. Heisler, M. D., Philadelphia.

Pathological Technique. By Frank B. Mallory, A.M., M.D., and James H. Wright, Harvard Medical School.

Diseases of Women. By J. Bland Sutton, F.R.C.S., London, and Arthur E. Giles, M.D., B.Sc., Lond., F.R.C.S., Edinboro.

---

The Twenty-fifth Annual Meeting of the American Public Health Association will be held at Philadelphia, Pa., October 26, 27, 28, 29, 1897.

The Executive Committee have selected the following topics for consideration: 1. The Pollution of Water-Supplies. 2. The

Disposal of Garbage and Refuse. 3. Animal Diseases and Animal Food. 4. Car Sanitation. 5. Steamship and Steamboat Sanitation. 6. The Prevention of the Spread of Yellow Fever. 7. The Transportation and Disposal of the Dead. 8. The Relation of Forestry to Public Health. 9. Nomenclature of Diseases and Forms of Statistics. 10. Cause and Prevention of Infectious Diseases. 11. Public Health Legislation. 12 Cause and Prevention of Infant Mortality. 13. Transportation of Diseased Tissues by Mail. 14. River Conservancy Boards of Supervision. 15. The Period during which each Contagious Disease is Transmissible, and the Length of Time for which each Patient is Dangerous to the Community. 16. Sanitation, with Special Reference to Drainage, Plumbing and Ventilation of Public and Private Buildings. 17. Some Method of International Arrangement for Protection against the Transmission of Infectious Diseases. 18. Disinfectants. 19. Existing Sanitary Municipal Organizations of the Countries belonging to the Association, with a view of a Report upon those Most Successful in Practical Results.

An announcement will be made in ample time before the meeting, giving full particulars regarding reduced fares on railroads, hotel rates and accommodations, special entertainments to be arranged by the local committee, etc.

All communications relating to local matters should be addressed to Dr. Benjamin Lee, Chairman Local Committee of Arrangements, No. 1532 Pine St., Philadelphia, Pa.

Following are the officers of the Association: President, Dr. Henry B. Horlbeck, Charleston, S. C.; First Vice-President, Dr. Peter H. Bryce, Toronto, Ont.; Second Vice-President, Dr. Ernest Wende, Buffalo, N. Y.; Secretary, Dr. Irving A. Watson, Concord, N. H.; Treasurer, Dr. Henry D. Holton, Brattleboro, Vermont.

